# Analysing extraction uniformity from porous coffee beds using mathematical modelling and computational fluid dynamics approaches

**DOI:** 10.1371/journal.pone.0219906

**Published:** 2019-07-31

**Authors:** Kevin M. Moroney, Ken O’Connell, Paul Meikle-Janney, Stephen B. G. O’Brien, Gavin M. Walker, William T. Lee

**Affiliations:** 1 Synthesis and Solid State Pharmaceutical Centre (SSPC), Bernal Institute, University of Limerick, Limerick, Ireland; 2 MACSI, Department of Mathematics and Statistics, University of Limerick, Limerick, Ireland; 3 Department of Chemical Sciences, University of Limerick, Limerick, Ireland; 4 Dark Woods Coffee, Holme Mills, West Slaithwaite Road, Marsden, Huddersfield, United Kingdom; 5 University of Huddersfield, Huddersfield, United Kingdom; University of New South Wales, AUSTRALIA

## Abstract

Achieving a uniform extraction of soluble material from a porous matrix is a generic problem in various separation and filtration operations, with applications in the food processing, chemical and pharmaceutical industries. This paper describes models of fluid flow and transport of soluble material within a packed granular bed in the context of coffee extraction. Coffee extraction is described by diffusion of soluble material from particles of one or more representative sizes into fluid flowing through the packed bed. One-dimensional flow models are compared to computational fluid dynamics (CFD) models. A fine and a coarse coffee grind are considered. Model results are compared to experimental data for a packed cylindrical coffee bed and the influence of a change in geometry to a truncated cone is considered. Non-uniform flow in the truncated cone causes significant variation in the local extraction level. Coffee extraction levels during brewing are analysed using extraction maps and the degree of variation is represented on the industry standard coffee brewing control chart. A high variation in extraction yield can be expected to impart bitter flavours into the brew and thus is an important variable to quantify.

## Introduction

Coffee is a globally important trading commodity. World coffee consumption is steadily increasing with published figures having reached over 157 million bags (9.42 × 10^9^ kg) in the year October 2016–September 2017 [1]. Brewed coffee is consumed in various forms, but there is a trend towards speciality coffee [2], placing a sharper focus on the quality of the product served to consumers. The demand on coffee appliance manufacturers to engineer a precise and reproducible process into their products is ever increasing. This has driven scientific investigation of all aspects of coffee production, from raw coffee cherries to the end beverage. Recent publications on roasting, degassing of coffee beans and grains, grinding and extraction include refs. [3–7]. Despite this, the goal of providing brewers with a general guide on how to control coffee quality for variations in conditions (coffee origin, water quality, grind size, flow rates, temperature, roast level etc.) remains elusive.

If you give the same coffee to ten baristas, you will end up with ten different tasting drinks. Years ago this inconsistency was put down to the “art” of making espresso. These days it is more likely to be attributed to the “science” of making espresso. The change in terminology and application of a scientific approach was largely initiated by Lockhart [8] and the Coffee Brewing Institute in 1957. However, this research related to filter coffee rather than espresso and so it was not part of the training of most baristas until relatively recently.

**Fig 1:**
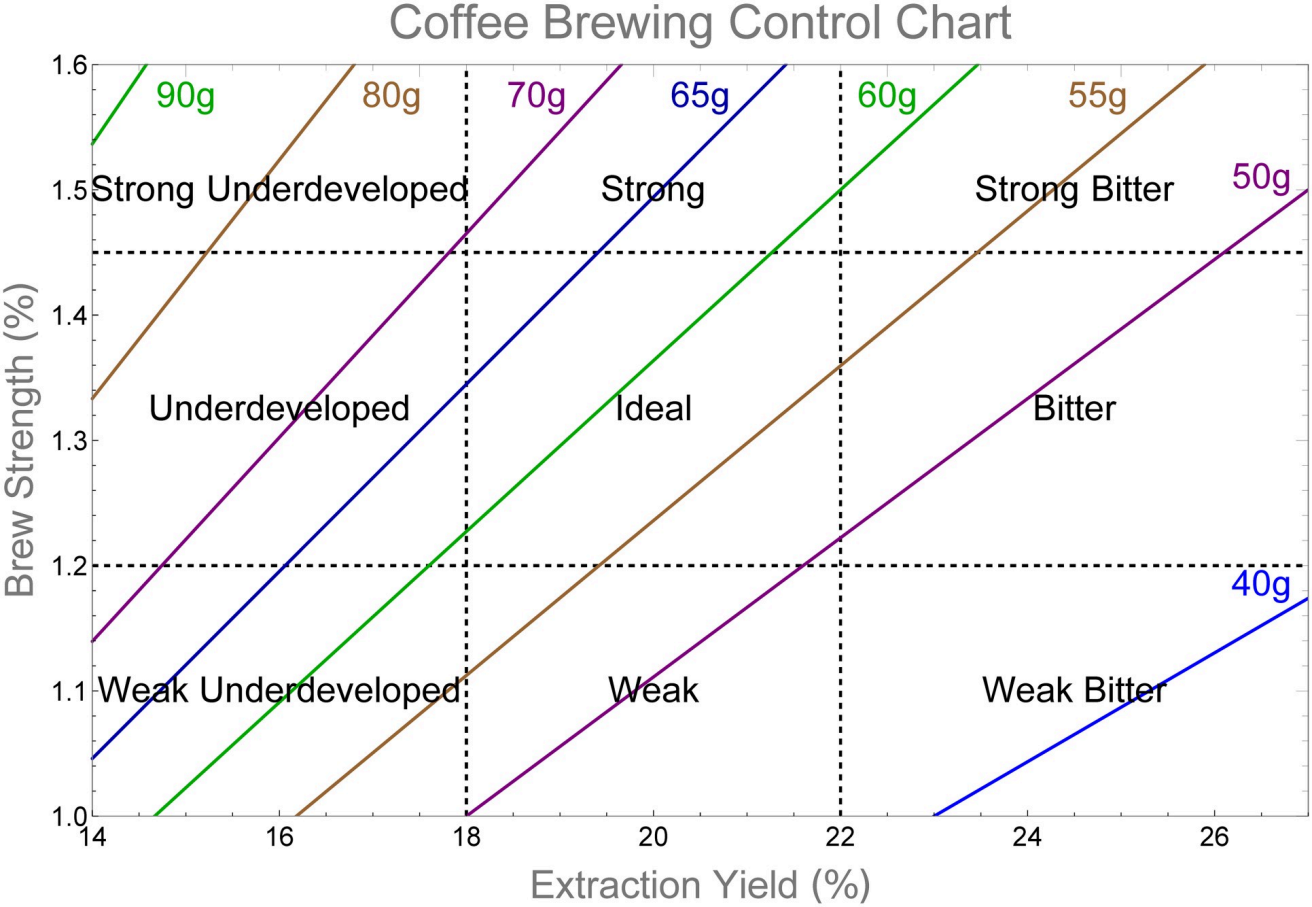
Coffee brewing control chart. Coffee brewing control chart example for drip filter coffee. The coffee quantities marked are for use with 1 kg of water.

At the heart of Lockhart’s research was the coffee brewing control chart. An example of such a chart is shown in Fig 1. The original chart defined a quantitative measure of coffee quality based on surveys of the American public’s preferences for coffee flavour and strength. The coffee brewing control chart represents brewed coffee flavour (quality) as a point on a chart of brew strength vs. extraction yield. The chart is divided into nine regions based on specified lower and upper limits for each variable. The vertical axis corresponds to brew strength (total dissolved solids (TDS)) and simply measures the mass concentration of dissolved coffee solids. For drip filter coffee beverages, brew strengths in the range 1.2%–1.45% (dependent on consumer geographic region) are considered ideal. Strength level is also a matter of cultural preference. The extraction yield, plotted on the horizontal axis, is considered the key measure of coffee flavour. It is the percentage of the dry coffee bed mass extracted into the beverage. Consumer taste preferences from the original research and a number of studies since suggest a region of optimal flavour at an extraction yield of 18%–22%. These two specifications overlap to produce a box in the centre of the chart that indicates the most desirable cup of filter coffee. For baristas this was seen as a target, as they tried to “brew in the box”. Recent research from the Speciality Coffee Association of Europe’s Gold Cup research program broadly confirmed this range [9]. This research ascertained consumer preferences in four different European cities though blind tastings of coffee with identical strength but different extraction yields.

The coffee brewing control chart was for filter coffee and so the recommended strength levels hold little relevance for the more concentrated espresso. Espresso brew strengths are much higher, with ranges of 8%–12% being typical. While there is no universal rule, concentrations of 5%–8% and 12%–18% have been categorised as the lungo and ristretto variants of espresso respectively by ref. [10]. Despite this, the same extraction yield percentage proved to be vital in espresso brewing.

Coffee has a multitude of dissolvable components imparting different flavours. Simplified, these have been grouped into acidic, sweet and bitter compounds, that dissolve at different rates throughout the extraction of an espresso. This means that the first part of the espresso is dominated by acidic/sour flavours, sweetness in the middle and bitterness towards the end. Hence, if a barista can control the quantity of different compounds dissolved from the coffee, they can control the balance of flavours. In simple terms it’s much like making a cup of tea, taking care not to “stew” it by brewing it for too long. The 18%–22% extraction yield, previously advised by Lockhart for filter coffee, is now broadly accepted as a target for a “balanced” espresso as well. Extraction yields under 18% (under-extracted) lack flavour and the coffee can taste too acidic. For extraction yields above 22% (over-extracted) the coffee can taste too bitter.

Baristas can now measure the extraction yield percentage through careful weighing of the mass of coffee used to make the espresso (the dose) and the mass of the espresso produced (the yield). Brewing recipes are thus typically given in terms of weight in (dose) and weight out (yield) (e.g. 18 g in –36 g out) along with the recommended shot time (the time it takes water to pass through the coffee bed). The shot time is controlled by grinding the coffee finer or coarser to increase or decrease the shot time respectively. A digital refractometer is used to measure the TDS in the espresso liquid. The extraction percentage can then be worked out by the following equation:

$$\frac{\text{Yield}\times \text{TDS}}{\text{Dose}}=\text{Extraction}\;\text{yield},$$ (1)

where yield and dose are typically in grams and TDS and extraction yield are percentages.

The coffee brewing control chart, while obviously useful, is often criticised as being overly simplistic. It gives a desired outcome, but gives no information on how to reach it, other then a recommended brew ratio (mass of dry coffee/mass of water used). A recent publication by Melrose et al. [11] suggests a new brewing control chart for extraction from a packed bed. The new chart connects the extraction yield and strength to measurable brewing conditions such as the coffee bed mass, water flow rate, particle size distribution and other factors. The chart is based on a physical model of extraction for the given grind distribution and bed porosity under consideration. It incorporates dimensionless numbers describing the bed extraction efficiency and the diffusive timescale for extraction from fine particles. The approach represents a step forward. However, one aspect of extraction neglected by both charts is a measure of extraction uniformity. Implicit in both brewing charts is the assumption that extraction is uniform within the coffee bed (or that any non-uniform extraction does not impact taste). Non-uniform extraction may occur due to inhomogeneous flow patterns, dead zones in the flow, agglomeration of fine particles or, in the worst case, regions of dry coffee in the bed. Development of flow channels (channelling), in shallow or poorly tamped beds, is a particular problem baristas have to avoid when brewing espresso coffee. Clearly at some level such non-uniformity will impact the flavour profile. One aim of this paper is to develop methods to quantify the variation of extraction yield within the bed.

Consideration of the variation in extraction yield within a packed coffee bed requires two main components. Firstly, a description of the extraction of soluble coffee species at the grain scale from which the local extraction yield can be calculated. The second requirement is a description of the fluid flow within the bed. The flow equations should be resolved in three spatial dimensions (or two, exploiting symmetry) to consider flow phenomena which occur within the bed. While a significant body of work exists on the chemistry of coffee, only in recent years has significant attention been given to modelling various physical aspects of coffee processing. A number of authors reported on the release kinetics of individual species and total soluble coffee content into dilute solutions. Initial work by Voilley and Simatos [12] considered the response of brew strength to variations in process parameters for well mixed systems of coffee grounds and water. Spiro and co-workers [13–17] reported extensive work on the release of caffeine from coffee grains into solution. These early bodies of work fitted their data using models for diffusion out of a sphere. Lee et al. [18] considered the release kinetics of a wide variety of molecular species using experiments which pumped water through a coffee basket at different rates. The extraction rate was quantified by the species half-life and the time to reach 90% extraction. Based on this, the molecular species were grouped into fast extractors and slow extractors. Since then a whole range of papers has quantified extraction of total soluble material and individual components from coffee grains and beds under various conditions [5, 7, 19–25].

A number of models have been developed which couple flow and extraction in coffee beds. Fasano et al. [26–29], developed general multiscale models for the extraction of coffee, primarily focused on the espresso coffee machine. In ref. [26] in particular, a comprehensive model of filtration in a multispecies porous medium is presented, which includes both mechanical and chemical interactions between the flow and the solid porous matrix. General equations are listed for transport of water and different species in the medium as either solids or solutes. The accumulation of migrating particles at the filter exit is also modelled. A system of partial differential equations is formulated and the existence and uniqueness of solutions to the problem is shown under certain conditions. The developed model is comprehensive, but the form of many of the terms governing mass transfer is not specified and no connection is made with experimental data. In recent years, a number of authors has developed models of the physics of coffee extraction and compared results with experimental data. Over a series of papers, Moroney et al. [30–33] developed and analysed a multiscale model of extraction of soluble material from packed coffee beds. Flow was described by Darcy’s law. Extraction followed first order rate equations describing extraction of coffee from two domains: easily accessible coffee in fine grains and the broken surfaces of larger grains and less accessible coffee in the intact cells in the kernels of larger grains. The models were validated by comparing their results experiments on extraction from a dilute suspension of coffee grains and a packed cylindrical bed. Melrose et al. [11, 34, 35] have applied similar models from a macroscopic standpoint to model flow and extraction in packed beds. A distinguishing feature of Melrose’s work, compared to ref. [30], is the use of models of diffusion in a sphere to describe extraction of coffee from particles of different sizes. Populations of large and small particles are used to represent the particle size distribution. Observed fast and slow extraction rates arise from the different particle sizes and the concentration profiles in the grains. The use of two diffusion coefficients to represent the extraction of fast and slow components is also considered. Kuhn et al. [7] studied extraction of caffeine and trigonelline from espresso beds. They fitted extraction by averaging material balance equations similar to those in ref. [11], first over the coffee particle diameter and then over the coffee bed height. The resulting model is spatially uniform and describes transport of coffee between the grains and the bulk fluid using first order rate equations. To ensure closure of the system a linear concentration profile is assumed in the liquid phase. Despite its simplified form relative to the models in refs. [11, 30] it is observed to fit the extraction profiles of caffeine and trigonelline reasonably well. Thus, significant progress has been made on modelling coffee extraction from cylindrical packed beds, but none of the work in the literature considers the spatial uniformity of flow and extraction in coffee beds in detail. This clearly should depend on the bed geometry and boundary conditions among other factors.

In this paper, we consider brewing coffee from a packed bed of coffee grains, which can include espresso and drip filter methods. Using recently developed models of coffee extraction and leveraging the power of established computational fluid dynamics (CFD) methods, we consider a neglected aspect of coffee quality, that of extraction uniformity in the coffee bed. This is a generic problem for any solid-liquid extraction or filtration operation where the target is to achieve uniform extraction and thus is equally useful in other contexts. The aims of the paper are to highlight the importance of extraction uniformity in brewing and to illustrate the power of CFD methods to investigate this for equipment design. A simple description of coffee extraction at the grain scale will be coupled with a spatially resolved flow model. The influence of non-uniform flow, generated by water delivery and bed geometry, on espresso type extraction will be considered. Such models can be used to investigate the impact of different brewing set-ups on extraction uniformity. The use of commercial CFD software allows generation of extraction maps, and has potential for virtual design of coffee equipment. This is the first step towards two-phase (water and coffee) and three-phase (water, coffee and gas) models of coffee brewing in realistic geometries.

## Experimental set-up

This section briefly outlines experimental work on coffee extraction conducted by Philips Research, Eindhoven. Some of the experiments have been detailed in ref. [30], but a more comprehensive description is provided here. The set-up for brewing coffee using a cylindrical chamber is detailed. The particle size distribution of a coarse and finely ground coffee is also characterised.

### Experimental rigging

**Fig 2:**
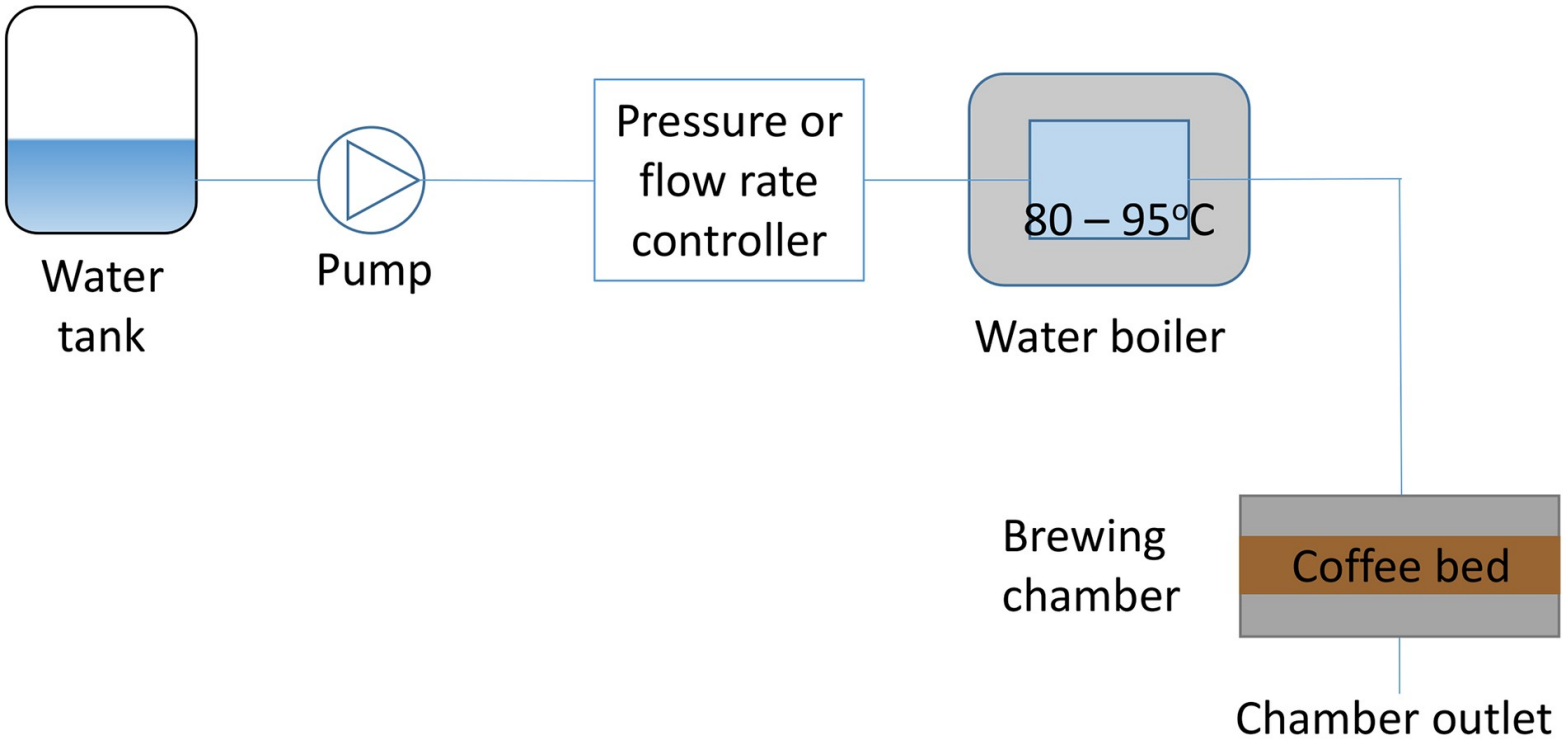
Experimental set-up. Schematic of the experimental set-up.

Experiments were designed to replicate a typical scenario for brewing coffee using a cylindrical type espresso brewing chamber. The experimental set-up is illustrated in Fig 2.

Water was pumped through the system using a Procon series-3 rotary vane pump, which is capable at delivering water pressures up to 16 bar. A 2 kW boiler was used to heat the water up to 90°C prior to pumping the water through the selected brewing chamber. The cylindrical stainless steel brewing chamber had a constant internal diameter of 59 mm. The bed height was permitted to change with respect to the volume of coffee inserted.

The water was introduced to the cylindrical coffee bed through a stainless steel sieve plate, ensuring a homogeneous irrigation. The system shown in Fig 2 can operate in a constant flow mode with the pressure differential across the bed adjusting itself to maintain the specified flow rate. Alternatively, the pressure differential across the coffee bed can be fixed, with the flow adjusting itself to the pressure through the system. For the experiments presented here, a constant flow rate of 250 ml min^−1^ was selected and the pressure drop across the bed was recorded. A dry coffee mass of 60 g was used for each tested case with various grind sizes. A total water volume of approximately 1 l was delivered for each extraction. It is noted that the extraction set-up is typical of an espresso extraction, but the quantities of coffee and water used and the total brewing time are more typical of drip filter extraction.

### Data acquisition and sensors

The extracted soluble coffee was routinely measured using a PAL3 Atago pocket refractometer. This produced a Brix number which linearly corresponded to the concentration of extracted coffee solubles. It was found that 1° Brix corresponded with a coffee concentration of 8.25 g l^−1^ as detailed in ref. [30]. The pressure drop across the system was monitored using a Bronkhorst P-502C electronic pressure transducer. A Bronkhorst CORI-FLOW mass flow meter was also used in-line to monitor the throughput of water. Both sensors allowed the control of either a constant pressure drop or constant flow rate when connected to a Bronkhorst C5I controllable valve.

### Coffee types

**Fig 3:**
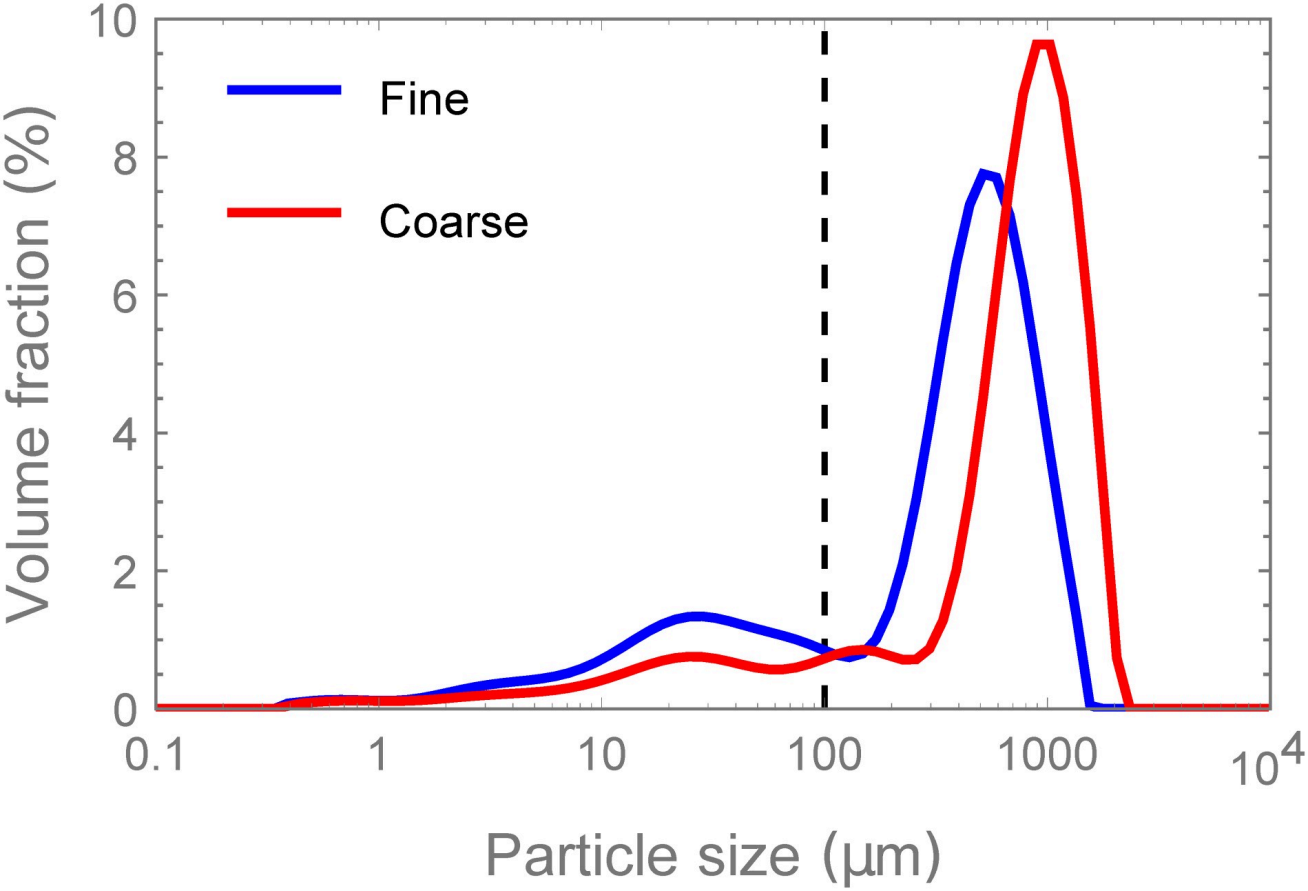
Coffee particle size distributions. Particle size distribution for both fine and coarse grinds.

A fine and a coarse coffee grind were selected for this study. The fine grind was a standard, unsieved Jacobs Krönung (JK) dripfilter grind. The coarse grind was obtained by grinding Illy coffee beans with a Cimbali burr grinder at setting #20. The mean diameters by area (Sauter mean diameter) and volume are reported in Table 1. The percentage of fines (particles with diameter < 100 μm here) is also reported. The exact choice of fines cut-off point is somewhat arbitrary. The 100 μm level chosen here, corresponds roughly to the local minimum typically found between the peaks of large and small particles observed in coffee grain particle size distributions after grinding. This level was also adopted in refs. [11, 35]. The coffee grind size plays a key role in both the pressure drop across the brewing chamber and the extraction rate from the coffee grinds in question. Smaller grain sizes permit the water to easily access the soluble coffee within the pores in comparison to the larger particles, which have intact cells enclosing the soluble coffee. The particle size distribution (PSD) for both coffee grinds selected is shown in Fig 3.

**Table 1:** Fine and coarse grind particle size distribution measures.

	*d*_3,2_	*d*_4,3_	Volume % < 100 μm
Fine	27.34 μm	457.84 μm	25.52
Coarse	37.78 μm	823.02 μm	15.08

It is evident that both grinds exhibit a bimodal distribution typical for ground coffee (although the coarse grind might be considered trimodal here). Thus, while we refer to the grinds as “fine” and “course”, each grind is really composed of a mixture of fine and coarse particles typical of many grinding processes. There is a significant peak at the desired grind size, but also a second peak occurs at a much smaller particle size. This second peak comprises small flakes, formed by fragments of coffee cell walls which fracture during grinding. This smaller peak occurs around 20μm to 40μm, which is the typical size of a coffee cell. These smaller particle sizes (fines) will contribute to a faster coffee extraction, being easily accessible to water and having a short diffusion distance. The fines will also allow a closer bed packing and provide a larger surface area to resist water flow. The coupling of a more tortuous path and higher surface resistance results in a larger pressure requirement to maintain a given flow rate.

## CFD coffee model

This section describes the approach taken to develop a numerical model using computational fluid dynamics (CFD) methods. Equations to describe the liquid phase are presented. A soluble coffee extraction model is detailed and coupled to a CFD flow model. Set-up and schematics of the simulation domain are listed for four test cases.

### Numerical model development

The Navier-Stokes equations, implemented within the commercially available ANSYS Fluent software, are used to solve the fluid flow problem within the computational domain. Multi-phase flow is modelled using the Euler-Euler equations. In this work, we assume a packed bed of coffee, which permits the velocity of the solid to remain zero (${\vec{v}}_{s}=0$). We also assume the volume fraction of the solid remains constant throughout the simulations. Assuming incompressible flow for the liquid phase gives the following mass conservation equation:

$$\nabla \cdot {\vec{v}}_{l}=0,$$ (2)

where ${\vec{v}}_{l}$ is the velocity of the liquid phase.

The numerical method solves for the motion of the liquid and solid phases separately. In this instance the solid phase solution is trivial. The phases are tracked explicitly. The volume of the liquid phase, *V*_*l*_, is given by:

$$V_{l}=\int_{V}^{}\alpha_{l}dV,$$ (3)

where *V* is the domain volume and the volume fraction of the liquid phase, *α*_*l*_, is tracked. The volume fractions of all phases must sum to one. Assuming steady state flow, the momentum equation is given by:

$$\alpha_{l}\rho_{l}\nabla \;\cdot \left({\vec{v}}_{l}{\vec{v}}_{l}\right)=-\alpha_{l}\nabla \;p_{l}+\alpha_{l}\rho_{l}\vec{g}+{\vec{F}}_{drag},$$ (4)

where the drag force interaction, ${\vec{F}}_{drag}$, from the solid granular phase is included. The drag is modelled through Eq (5):

$${\vec{F}}_{drag}=K_{sl}({\vec{v}}_{s}-{\vec{v}}_{l}).$$ (5)

The drag force interactions are modelled using the Gidaspow model [36]. In cases where the volume fraction of the solid phase, *α*_*s*_ is less than 0.8, the following is employed:

$$K_{sl}=150\frac{\alpha_{s}(1-\alpha_{l})\mu_{l}}{\alpha_{l}d_{s}^{2}}+1.75\frac{\rho_{l}\alpha_{s}|{\vec{v}}_{s}-{\vec{v}}_{l}|}{d_{s}},$$ (6)

where *μ*_*l*_ is the viscosity of the fluid, *d*_*s*_ is the diameter of the solid and *ρ*_*l*_ is the density of the liquid phase. The first term in Eq (6) corresponds to the commonly used Kozeny-Carman description of permeability for a bed of spherical particles. This expression can be utilised for each granular phase to include more than one grain size as required.

As mentioned above, the coffee bed is assumed to be sufficiently packed to ensure no grain movement occurs. This reduces the complexity of the problem and the resulting model is close to Darcy’s law in the liquid phase for the velocities considered. We do not consider any stress build up in, or compression of, the solid phase which only provides a momentum sink for the liquid phase.

### Coffee extraction model

This section describes the model for extraction of coffee solubles by the water from the grains. The interfacial area of per unit volume of the coffee grains, *A*_*i*_, is calculated by:

$$A_{i}=\alpha_{s}A_{s}=\frac{6\alpha_{s}}{d_{s}},$$ (7)

where *A*_*s*_ is the surface-to-volume ratio of a grain with diameter *d*_*s*_. This is based on the assumption of spherical grains. In reality, grains deviate somewhat from perfect spheres, with sphericity values of 0.75–0.8 [11, 35] and ∼ 0.75–0.85 [7] (depending on grain size) reported in the literature. Sphericity of a particle refers to the ratio of the surface area of a sphere of the same volume as the particle, to the actual surface area of the particle. For simplicity, we assume spherical particles here.

The transfer of soluble coffee from the coffee grinds to the water is modelled through the following equations:

$$\frac{\partial }{\partial t}(c_{l}\alpha_{l})+\nabla \;\cdot ({\vec{v}}_{l}c_{l}\alpha_{l})=h_{sl}A_{i}(c_{s}-c_{l}),$$ (8)

$$\frac{\partial }{\partial t}(c_{s}\phi_{v}\alpha_{s})=-h_{sl}A_{i}(c_{s}-c_{l}),$$ (9)

where *c*_*s*_ and *c*_*l*_ are the concentration of coffee in the grains and intergranular pores respectively. The mass transfer coefficient of soluble material from the grind to the passing liquid is denoted *h*_*sl*_. We assume both volume fractions and the intragranular porosity, *ϕ*_*v*_, remain constant. Through this, we can rearrange Eqs (8) and (9) and substitute (7) to yield:

$$\frac{\partial c_{l}}{\partial t}+\nabla \;\cdot (\vec{v}c_{l})=\frac{6h_{sl}\alpha_{s}}{\alpha_{l}d_{s}}(c_{s}-c_{l}),$$ (10)

$$\frac{\partial c_{s}}{\partial t}=-\frac{6h_{sl}}{\phi_{v}d_{s}}(c_{s}-c_{l}).$$ (11)

These extraction equations are incorporated into the CFD model developed in the following section. The initial concentrations of soluble coffee within the grinds are obtained using the following equation:

$$c_{s0}=\frac{\phi_{0}\rho_{s}}{\phi_{v}}.$$ (12)

This is based on the volume fraction of soluble coffee, *ϕ*_0_, the intragranular porosity, *ϕ*_*v*_, and coffee true density, *ρ*_*s*_. Alternatively it may be determined using the maximum theoretical extractable yield. This model assumes that particles are initially wet and all soluble coffee is dissolved in the intragranular pore space. Thus *c*_*s*_ is representative of the actual (grain averaged) concentration in the intragranular pore space and no partition coefficient is used. Melrose et al. [11] suggest a partition coefficient of 0.6 for their model which uses a concentration averaged over the whole grain volume and resolved with the grain radius. However when fitting their models they use a partition coefficient of 1.

Ref. [30] reported a coffee true density of 1400kg m^−3^ for the same coffees as used here and an intragranular porosity of *ϕ*_*v*_ = 0.56. For the two coffee types used in this paper, fine grind and coarse grind, the volume fractions of soluble coffee estimated in the respective grains are *ϕ*_0_ = 0.143 and *ϕ*_0_ = 0.122, based on experimental calculation of the maximum yields. The increased amount of extractable soluble material in finer grinds is a phenomenon widely noted in the literature [30, 35]. The description in Eq (12) of the initial soluble coffee content leads to values above a theoretical maximum saturation concentration (proposed in [30]), during the initial extraction phase. This is due to the incorrect assumptions of instantaneous wetting of grains and dissolution of soluble material into the pores. While recognising these limitations, this reduced model will be used as a reasonable base description on which to explore extraction uniformity. The resulting complexity savings mean we are only required to solve a system of two coupled PDEs, while still providing a reasonable fit of macroscopic soluble coffee concentrations.

In order to describe the system with an acceptable degree of accuracy, it will be necessary to account for at least two representative grain sizes. Recalling the PSD in Fig 3, two peaks are evident of a fine grain size and a coarse grain size, denoted by subscript 1 and 2, respectively. To include these in our system, the following equations are implemented:

$$\begin{array}{cc} \frac{\partial c_{l}}{\partial t}+\nabla \;\cdot (\vec{v}c_{l}) & =\frac{6h_{sl1}\alpha_{s1}}{\alpha_{l}d_{s1}}(c_{s1}-c_{l})\\ & +\frac{6h_{sl2}\alpha_{s2}}{\alpha_{l}d_{s2}}(c_{s2}-c_{l}), \end{array}$$ (13)

$$\frac{\partial c_{s1}}{\partial t}=-\frac{6h_{sl1}}{\phi_{v1}d_{s1}}(c_{s1}-c_{l}),$$ (14)

$$\frac{\partial c_{s2}}{\partial t}=-\frac{6h_{sl2}}{\phi_{v2}d_{s2}}(c_{s2}-c_{l}).$$ (15)

The generalisation to more then two representative grain sizes is straightforward. The parameters values associated with the coffee grains for both grinds are listed in Table 2.

**Table 2:** Coffee grain parameters for fine and coarse grinds for both single grain and two-grain CFD and 1-D models.

		Single grain	Two grain small	Two grain large
Fine	*d*_*s*_	3.1823 × 10^−5^ m	2.517 × 10^−5^ m	5.63 × 10^−4^ m
	*α*_*s*_	0.8	0.5	0.3
	*c*_*s*_	358.587 kg m^−3^	358.587 kg m^−3^	358.587 kg m^−3^
	*h*_*sl*_	4.31562 × 10^−4^ ms^−1^	5.1207 × 10^−4^ ms^−1^	1.43 × 10^−3^ ms^−1^
Coarse	*d*_*s*_	4.5802 × 10^−5^ m	3.536 × 10^−5^ m	9.26 × 10^−4^ m
	*α*_*s*_	0.75	0.45	0.3
	*c*_*s*_	305 kg m^−3^	305 kg m^−3^	305 kg m^−3^
	*h*_*sl*_	9.5285 × 10^−4^ ms^−1^	1.661 × 10^−4^ ms^−1^	2.901 × 10^−4^ ms^−1^

### Computational domain

**Fig 4:**
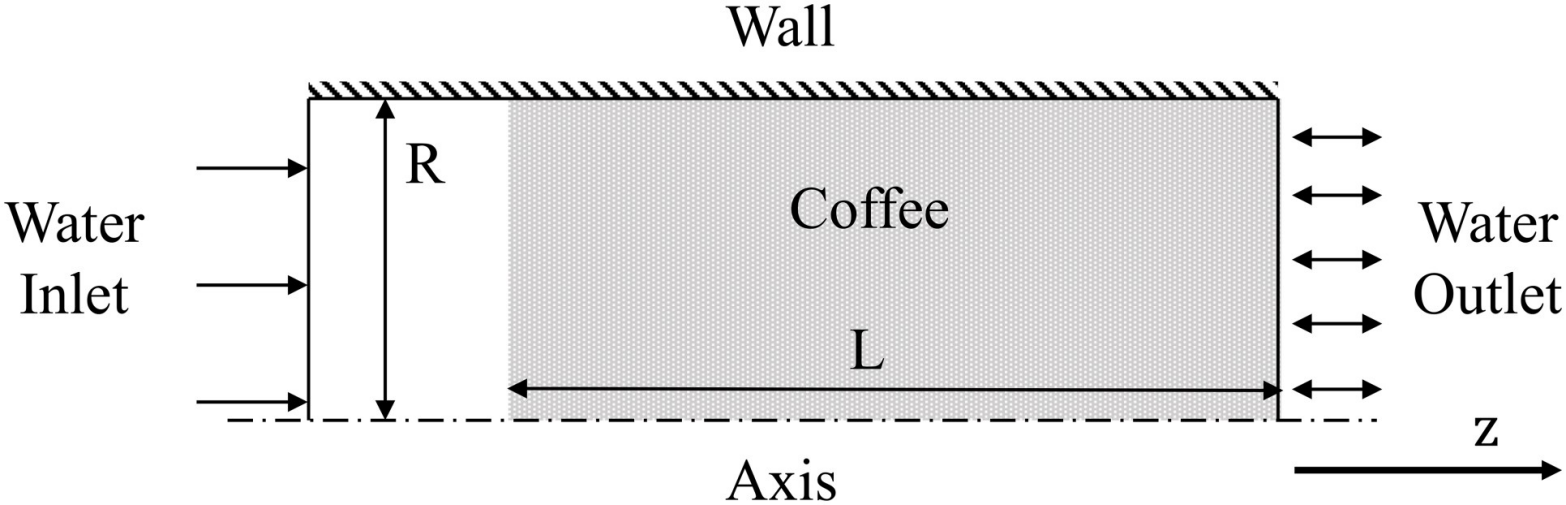
Cylindrical case computational domain. Cylindrical case computational domain with included boundary conditions.

The computational domain is modelled according to the experimental work described in Experimental set-up. A transient implicit time stepping method is employed here with time steps of 0.05s to ensure the flow through the domain is fully resolved. Residuals of equations are also required to drop at least three orders of magnitude to satisfy convergence requirements. The SIMPLE (Semi-Implicit Method for Pressure-Lined Equations) pressure-velocity coupling scheme is employed [36]. A packed bed espresso model is generated and axisymmetry is utilised to reduce computational efforts. A schematic of the espresso domain is presented in Fig 4.

The length of the coffee domain, *L*, was specified to match the experimental packed bed heights. Similarly, the radius of the espresso bed, *R*, was obtained from experimental set-up. The domain was discretised using a structured mesh of 4k cells.

The corresponding boundary conditions are presented in Fig 4 where the walls use a no-slip wall boundary and an axial symmetry condition is applied at the cylinder’s axis. A pressure outlet is employed to permit the liquid to escape the computational domain. The water inlet is incorporated using a mass flow boundary condition to specify the correct flow, calculated from the flow rate in the corresponding experiments.

**Fig 5:**
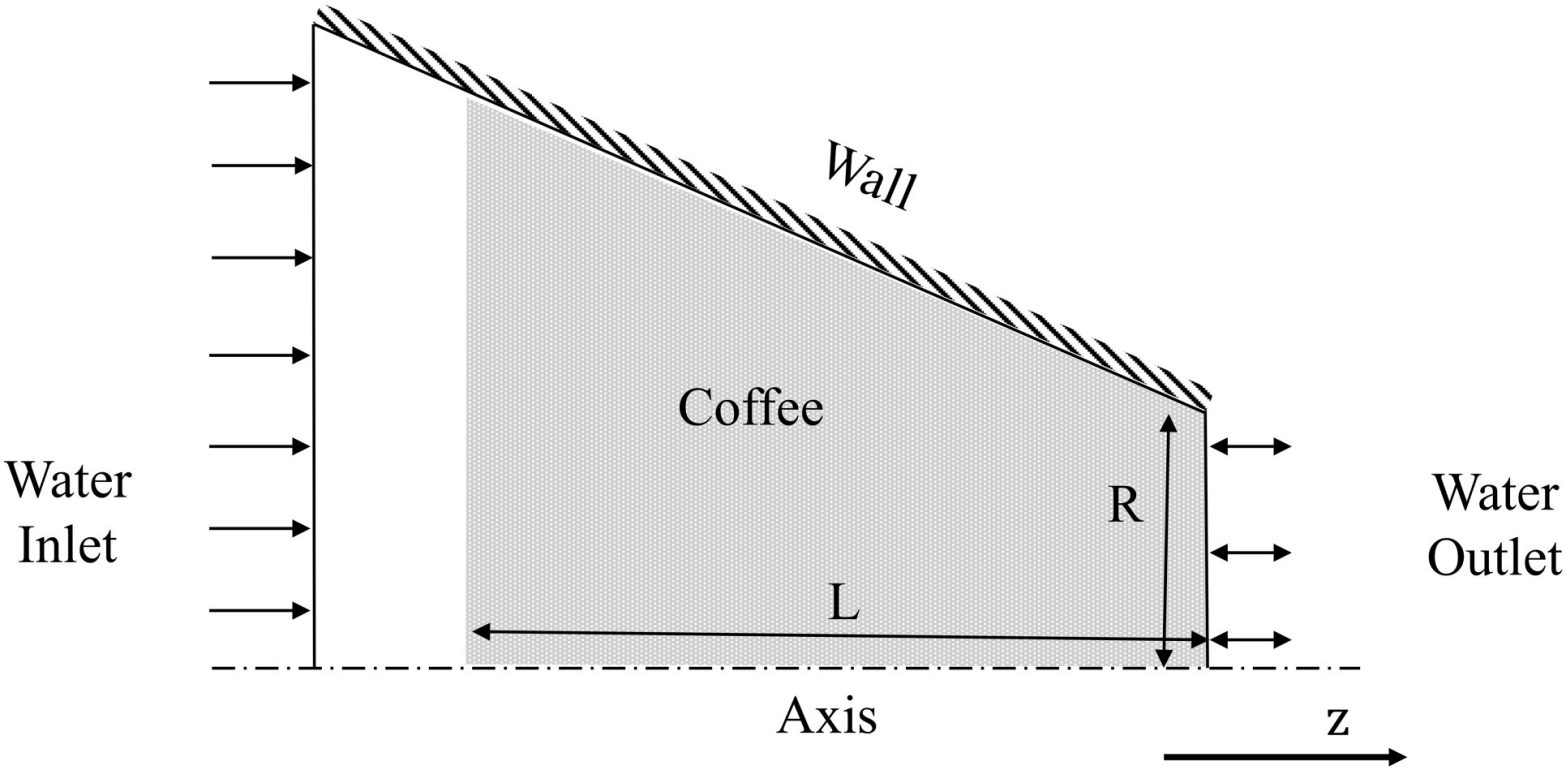
Conical case computational domain. Conical case computational domain with included boundary conditions.

A truncated cone geometry (similar to those used in pour-over or drip filter brewing methods) is also analysed through similar methods described above, using the same assumption of a packed bed. Fig 5 presents the computational domain used to consider the impact of a change of geometry on the extraction experiments carried out using a cylindrical vessel. A truncated cone with an apex angle of 60° was used. The opening radius at the truncation point, *R*, was set at 0.018 m. The coffee bed height, *L*, was then calculated to have the same volume of coffee grinds used in the cylindrical vessel. The same flow rate and boundary conditions as in the cylindrical vessel were enforced and the bed packing was assumed the same (independent of any pressure changes to maintain flow-rate).

The numerical methods to solve the flow for this case are similar to those used in the cylindrical case. A discretisation of approximately 24k cells using a structured mesh is employed here. A time step of 0.05 s, the same as the previous cylindrical case, is used to advance time and fluid flow. A mesh sensitivity study was conducted following the methods outlined in refs. [37, 38] to verify the accuracy of the numerical simulations. The coffee concentration at a fixed time was selected as the parameter to study. A refinement ratio of 2 was used resulting in coarser grids of 1.5k and 6k cells. Thus the normalised grid spacings are 1, 2 and 4 as the grids get coarser. Based on the approximated errors between mesh refinements an order of convergence *p* of 1.85 was calculated, close to second order convergence. Oscillatory convergence was observed with grid convergence indices (GCI) having very low values of GCI_2,3_ = 0.016% and GCI_1,2_ = 0.005%. The GCI values were calculated using a safety factor of 1.25 following ref. [38]. It was confirmed results are within the asymptotic range of convergence. Thus the selected discretisation is more then sufficient to ensure numerical accuracy.

## One-dimensional models

### 1-D model for cylindrical geometry

A one-dimensional model of coffee extraction in a packed bed is outlined here. This simplified model is used to compare with the more complex CFD simulations. Considering the cylindrical espresso coffee bed of cross-sectional area, *A*, and height, *L*, we obtain a bed domain of 0 < *z* < *L*, where *z* = 0 is the inlet and *z* = *L* is the outlet. Through the assumption of no change in solid density and a static bed, the mass and momentum equations to describe the solid bed are identically satisfied. No change in bed consolidation level is accounted for. In the liquid phase, Darcy’s law is used for the momentum equation while mass conservation results in the continuity equation. Following ref. [30], we reduce the general equations to one-dimensional flow by neglecting pressure gradients and flow velocity perpendicular to the axis of the cylinder. Thus only velocity and pressure variations in the *z* direction are modelled. We note the permeability *k*_*sl*_ commonly used in Darcy’s Law is related to *K*_*sl*_ by:

$$k_{sl}=\frac{\alpha_{l}^{2}\mu_{l}}{K_{sl}}.$$ (16)

For uniformity of presentation we adopt *K*_*sl*_ in further equations and describe it using Eq (6). The inlet boundary condition can be applied through a fixed pressure or fixed volumetric flux condition. Since we only consider steady state flow, these conditions are equivalent here. They are given by:

$$p_{l}\left(0,t\right)=\Delta p,\;p_{l}\left(L,t\right)=0,$$ (17)

and

$$v_{l}\left(0,t\right)=\frac{Q}{\alpha_{l}A},\;p_{l}\left(L,t\right)=0,$$ (18)

where Δ*p* is the pressure drop across the domain and *Q* is the volumetric flow rate through the system. Either a pressure or velocity boundary condition can be used at the inlet in this instance. The representative grain diameter is selected to ensure the pressure drop across the bed matches the liquid velocity. In the case of two grain sizes, one grain size is fixed from the particle size distribution and the other is used to fit the experimentally observed pressure drop. An alternative is to fix the granular sizes and choose the liquid volume fraction to match the pressure drop. Care should be taken here. The volume fraction available for flow may be larger than the true intergranular porosity since some of the grain surfaces may be permeable to water. Thus, the solutions to the one dimensional problem are:

$$v_{l}=-\frac{\alpha_{l}}{K_{sl}}(\frac{\Delta p}{L}-\rho_{l}g),$$ (19)

or

$$p_{l}=\frac{\Delta p}{L}(L-z).$$ (20)

In reality the volume fraction may change due to bed consolidation, coffee dissolution, swelling of the coffee cell walls or other physical or chemical transformations. It is unclear whether the net result of these competing processes is in an overall increase or decrease in the volume fraction available for flow during brewing. The qualitative result of a change in volume fraction can be seen from the extraction equations. For a fixed volumetric flow rate an increase in liquid volume fraction results in a decreased pore velocity and increased residence time ($\frac{\alpha_{l}AL}{Q}$). Although offset initially by an increased dilution capacity in the liquid phase, the reduced residence time would limit the extraction rate at later stages of extraction. A decrease in volume fraction has the opposite effects. For a fixed pressure difference across the bed an increase in volume fraction will lead to a higher permeability and pore velocity and a lower residence time. Thus the extraction rate will be higher even if the concentration in the liquid phase is lower. Again the opposite is true for a decreased liquid volume fraction under a fixed overpressure. In the absence of experimental insight the liquid volume fraction is assumed constant here. Hence, the coupling between the liquid flow equations and the extraction equations is uni-directional. The flow equations can thus be solved independently first and the resulting flow field applied to the extraction problem as required. For a single grain size, the soluble coffee content distribution within both the solid and liquid phase is resolved using the following equations:

$$\frac{\partial c_{l}}{\partial t}+v_{l}\frac{\partial c_{l}}{\partial z}=-\frac{1-\alpha_{l}}{\alpha_{l}}D_{v}\frac{6}{d_{s}^{2}}(c_{l}-c_{s}),$$ (21)

$$\frac{\partial c_{s}}{\partial t}=D_{v}\frac{6}{d_{s}^{2}}(c_{l}-c_{s}),$$ (22)

where *D*_*v*_ is the effective mass diffusivity of soluble coffee from the grains to the water. The two grain extraction equations are analogous to Eqs (13)–(15) (with $h_{sli}=\frac{D_{v}}{d_{si}}$, *i* = 1, 2).

$$\begin{array}{cc} \frac{\partial c_{l}}{\partial t}+v_{l}\frac{\partial c_{l}}{\partial z} & =\frac{6D_{v}\alpha_{s1}}{\alpha_{l}d_{s1}^{2}}(c_{s1}-c_{l})\\ & +\frac{6D_{v}\alpha_{s2}}{\alpha_{l}d_{s2}^{2}}(c_{s2}-c_{l}), \end{array}$$ (23)

$$\frac{\partial c_{s1}}{\partial t}=-\frac{6D_{v}}{\phi_{v1}d_{s1}^{2}}(c_{s1}-c_{l}),$$ (24)

$$\frac{\partial c_{s2}}{\partial t}=-\frac{6D_{v}}{\phi_{v2}d_{s2}^{2}}(c_{s2}-c_{l}).$$ (25)

Diffusion of dissolved coffee solids within the fluid phase is neglected since transport in this phase is advection dominated [30]. This means just one boundary condition at the inlet is required (zero concentration in the incoming water at the inlet):

$$c_{l}\left(0,t\right)=0.$$ (26)

Initially (at *t* = 0) the bed and grains are assumed fully wetted. The initial conditions of the liquid and of the grains can be described by the following:

$$c_{l}\left(z,0\right)=0,\;c_{s}\left(z,0\right)=c_{s0}.$$ (27)

The initial concentration, *c*_*s*0_, can be obtained using the previously defined Eq (12). This is applied in the same manner for both coffee types described in Experimental set-up. The model parameters are selected using data from both fine and coarse coffee experiments in the cylindrical brewing chamber given in Table 2. Mass transfer coefficients (effective diffusion coefficients) are fitted using the 1-D model and applied to both the 1-D and CFD models. The solutions of the 1-D model are compared to the experimental data and CFD simulations in the following section.

### 1-D model for truncated cone

In the conical case, an equivalent 1-D model was used with the equations being corrected using the cross-sectional area of the bed. This area was calculated as a function of the bed depth *z*. Pressure and velocity variations perpendicular to the conical axis were neglected. Thus increasing fluid velocity and dropping pressure parallel to the conical axis were accounted for. This assumption can only be expected to be very accurate for a small tapering of the walls. The coordinates 0 < *z* < *L*_*B*_ are considered where *z* = 0 is the inlet of the bed (top of cone) and *z* = *L*_*B*_ is the outlet. The theoretical cone vertex obtained extending the walls beyond the truncation point is at *z* = *L*_*c*_ (with *L*_*c*_ > *L*_*B*_). The radius and cross-sectional area at any position *z* with 0 ≤ *z* ≤ *L*_*B*_ are given by:

$$r(z)=\left(L_{c}-z\right)\text{tan}\;\theta_{c},\;A(z)=\pi {\left(L_{c}-z\right)}^{2}{\text{tan}}^{2}\;\theta_{c},$$ (28)

where *θ*_*c*_ is the half angle at the cone vertex. Assuming the same volumetric flow rate at the inlet as the cylindrical case, the pore velocity and pressure at a point *z* can be calculated as:

$$v_{l}\left(z\right)=\frac{Q}{\alpha_{l}\pi {\left(L_{c}-z\right)}^{2}{\text{tan}}^{2}\;\theta_{c}},$$ (29)

$$\begin{array}{cc} p_{l}\left(z\right) & =\frac{K_{sl}Q}{\pi \alpha_{l}^{2}{\text{tan}}^{2}\;\theta_{c}}(\frac{L_{B}-z}{\left(L_{c}-L_{B}\right)\left(L_{c}-z\right)})\\ & -\rho_{l}g\left(L_{b}-z\right). \end{array}$$ (30)

As above, due to the underlying assumptions of the model, the flow problem is independent of the extraction problem. The equations for coffee extraction are identical to those in Eqs (23)–(25), except Eq (29) replaces the constant velocity assumption in Eq (23). The boundary and initial conditions for the extraction problem are the same as the cylindrical case.

Both one-dimensional models were implemented numerically in MATLAB^®^ R2018a (MathWorks^®^). The system was solved using the numerical method of lines. The equations were discretised in space using first order backward differences for derivatives and solved forward in time using the inbuilt solver for stiff problems, ode23s. This solver is an implementation of an explicit Runge-Kutta (2,3) pair of Bogacki and Shampine [39, 40]. A sufficiently fine mesh was chosen to ensure no excessive numerical diffusion took place.

## Results and discussion

Simulations using the CFD methodology were completed using a high performance computer of 24 Xeon E5-2699 processors with 128GB of RAM. Initialisation of the domain in both cases was completed with a fully water saturated coffee bed. The water phase initially had zero velocity and a coffee concentration of zero. Soluble coffee content within the grains was initialised using values from Table 2. The results from both numerical models are presented in the cylindrical case and validated using experimental data. These numerical models were modified and applied to analyse the extraction rates of coffee in a conical geometry as specified above.

### Cylindrical case

The results from both the CFD and one-dimensional models are presented here for a cylindrical bed extraction. For the finely ground coffee bed, simulations were monitored and the pressure drop across the CFD computational domain was measured to be 2.319 bar. The 1-D model produced a pressure drop of 2.3 bar as expected as it was used to fit the experimentally observed relationship between flow velocity and pressure drop. Both numerical models produce excellent agreement to the experimentally recorded value of 2.3 bar. This indicates Darcy’s law is sufficient here to describe the flow through the cylindrical bed.

**Fig 6:**
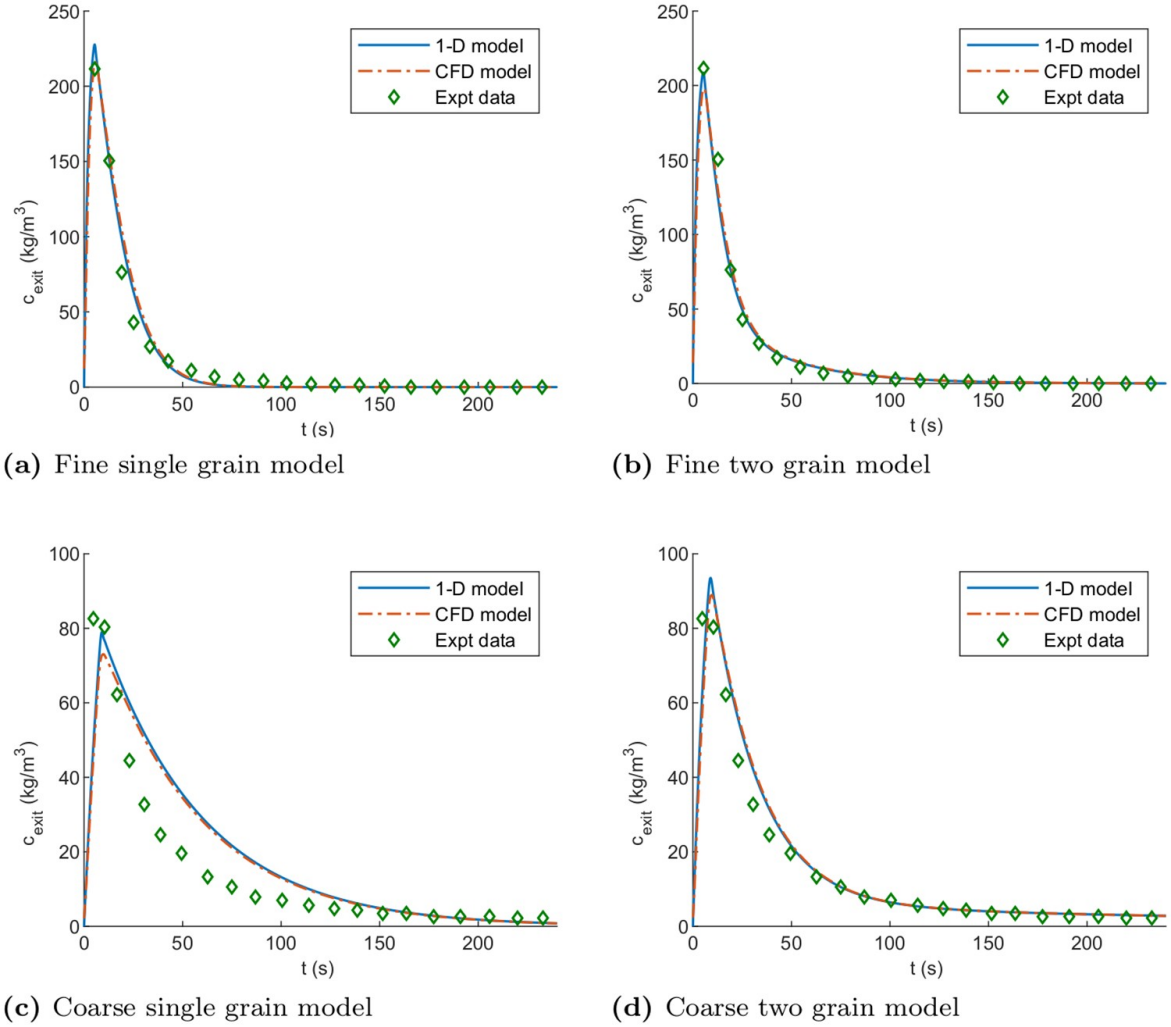
Numerical and experimental results cylindrical case. Comparison between experimental (Expt data) and numerical outlet coffee concentrations for different models for the fine grind ((a) and (b)) and the course grind ((c) and (d)).

The concentration of soluble coffee within the water and grain phases was monitored throughout both simulations. Plots of the concentration of soluble coffee within the water phase at the outlet (*c*_exit_) are presented in Fig 6(a) and 6(b). Experimental data is abbreviated to “Expt data” in the figure legends. Fig 6(a) shows the comparison of the models and experimental data for a single grain extraction representation. The single grain model is unable to capture the two rates of extraction evident in the concentration data. This is in agreement with previous work in the literature. In Fig 6(a), the initial fast extraction regime has been fitted, but it is clear the slower extraction tail cannot be captured.

Using a model incorporating a mixture of small and large grains to represent the grind size distribution, shows a much improved fit over the single grain approach. Fig 6(b) demonstrates that the two grain model can also capture behaviour in the region beyond the initial ∼ 40 s of extraction. The root mean squared error for the 1-D model decreases from 7.80 kg m^−3^ to 5.81 kg m^−3^.

The coarsely ground coffee bed, with parameters described in Table 2, is also simulated using both numerical models. These are further compared to the experimental work using the coarse grind. The comparison of the single grain and two grain representations to experiment are shown in Fig 6(c) and 6(d). The root mean squared error for the 1-D model decreases from 11.65 kg m^−3^ to 6.23 kg m^−3^ when the two grain model is used. The error is similar to that for the fine grind and largely arises from difficulty in accurately fitting the initial extraction period following bed saturation. The pressure drop across the bed within the CFD model was found to be 0.657 bar. The experimental measurement was recorded to be 0.65 bar across the brew chamber and this was used to calibrate the velocity-pressure relationship in the 1-D model. The CFD simulation showed the pressure profile to be linear within the coffee bed, corresponding to a uniform velocity and resistance of the bed, as assumed in the 1-D model.

Applying the two grain model, the results of both 1-D and CFD models show excellent agreement with experimental data. Thus, for either grind type, both the 1-D and CFD models, utilising a mixture of small and large grains, can reproduce the experimentally observed flow and extraction characteristics. As the flow field is essentially 1-D in this case, the increased modelling accuracy of the CFD model does not add significant benefit.

In terms of extraction uniformity, the only modelled variation is parallel to the bed axis (*z*-direction). The fresh water introduced at the bed inlet will maintain a higher concentration gradient between the grains and the interstitial solvent, indicating that extraction occurs fastest at the bed inlet and slowest at the outlet. The magnitude of this difference depends on the ratio of the volumetric flow rate into the bed and the volume of pore space in the bed. Larger differences will exist for lower flow rates and limited pore space during the initial periods of rapid extraction.

### Conical case

The impact of changing the bed geometry to a truncated cone (shown in Fig 5) is considered here. This geometry is similar to that commonly used in the pour-over method of making coffee. We consider the same coffee, a packed bed and the specified volumetric flow rate at the bed inlet from the cylindrical case. The resulting pressure drop and flow velocity profile is modelled using both the 1-D and CFD models described above. No change in bed packing due to consolidation is accounted for. Coffee extraction as influenced by the flow behaviour is considered.

**Fig 7:**
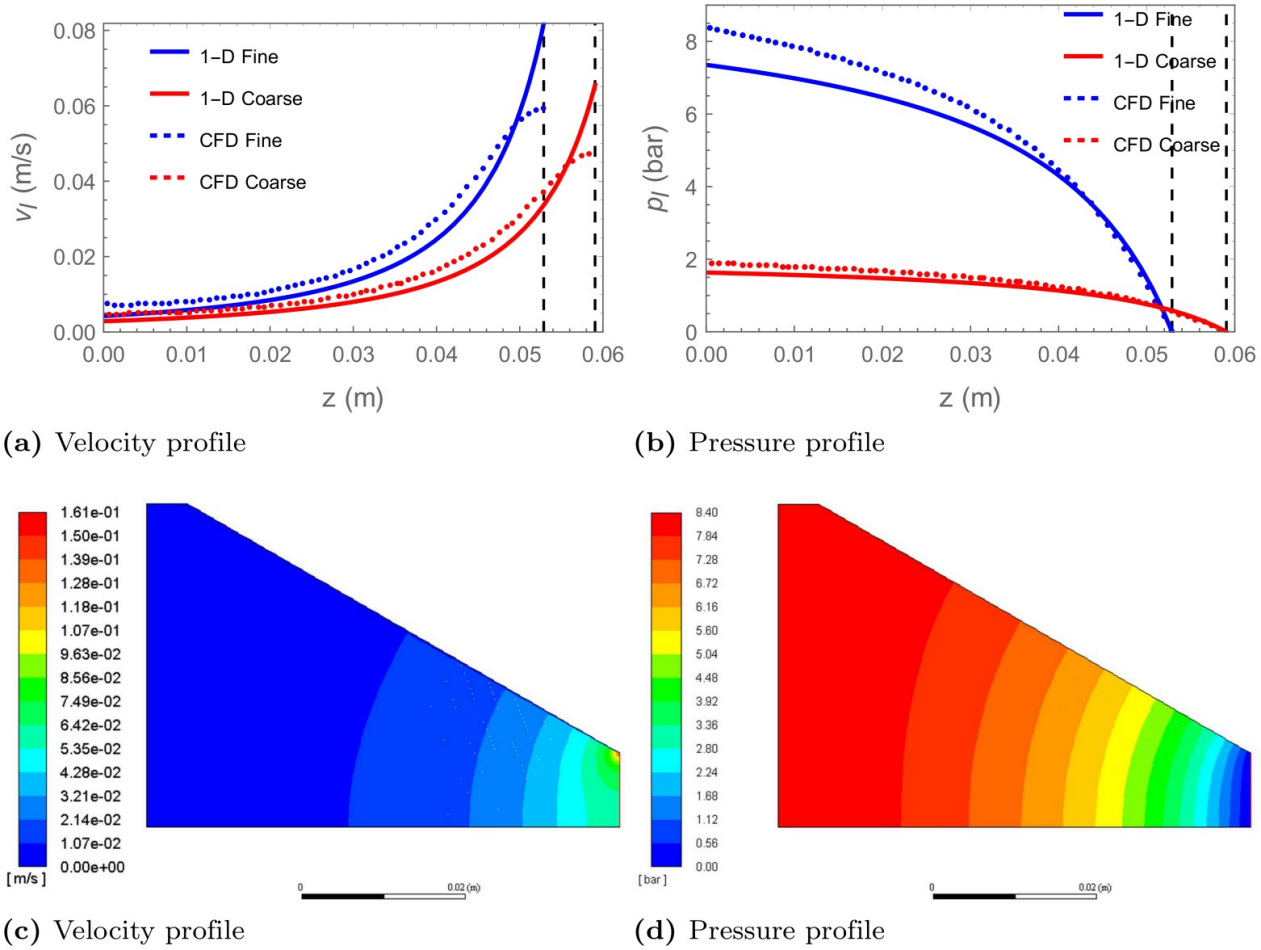
Liquid velocity and pressure profiles conical case. Velocity and pressure profiles of the liquid observed in the 1-D cone model (solid line) and in the CFD model at the centre line of cone (dashed lines) for fine and coarse grinds ((a) and (b)). Velocity and pressure profiles of the liquid observed for fine coffee within the CFD model ((c) and (d)).

The 1-D cone model predicts pressure drops of approximately 7.35 bar and 1.63 bar for the fine and the coarse grind respectively. This compares with values of 8.4 bar and 1.89 bar for the CFD simulations. This discrepancy between the CFD and 1-D results is expected because the 1-D model cannot truly describe the conical geometry. The velocity and pressure profiles are shown for the 1-D model and the centreline (cone axis) in the CFD model in Fig 7(a) and 7(b) respectively.

The velocity profile of the water as it flows through the static bed of the fine grind is presented in Fig 7(c). An uneven distribution is observed with a peak velocity occurring at the corner of the outlet and the cone wall. The pressure and velocity vary both parallel and perpendicular to the conical axis. The significant variation of the flow behaviour from the 1-D model, can be seen from the velocity and pressure fields, shown in Fig 7(d). Here, we see a uniform pressure at the inlet of the domain but as the walls narrow, the modelled pressure varies in both the axial and radial directions of the domain. Equivalent results, but with a smaller pressure drop, are observed for the coarse grind.

**Fig 8:**
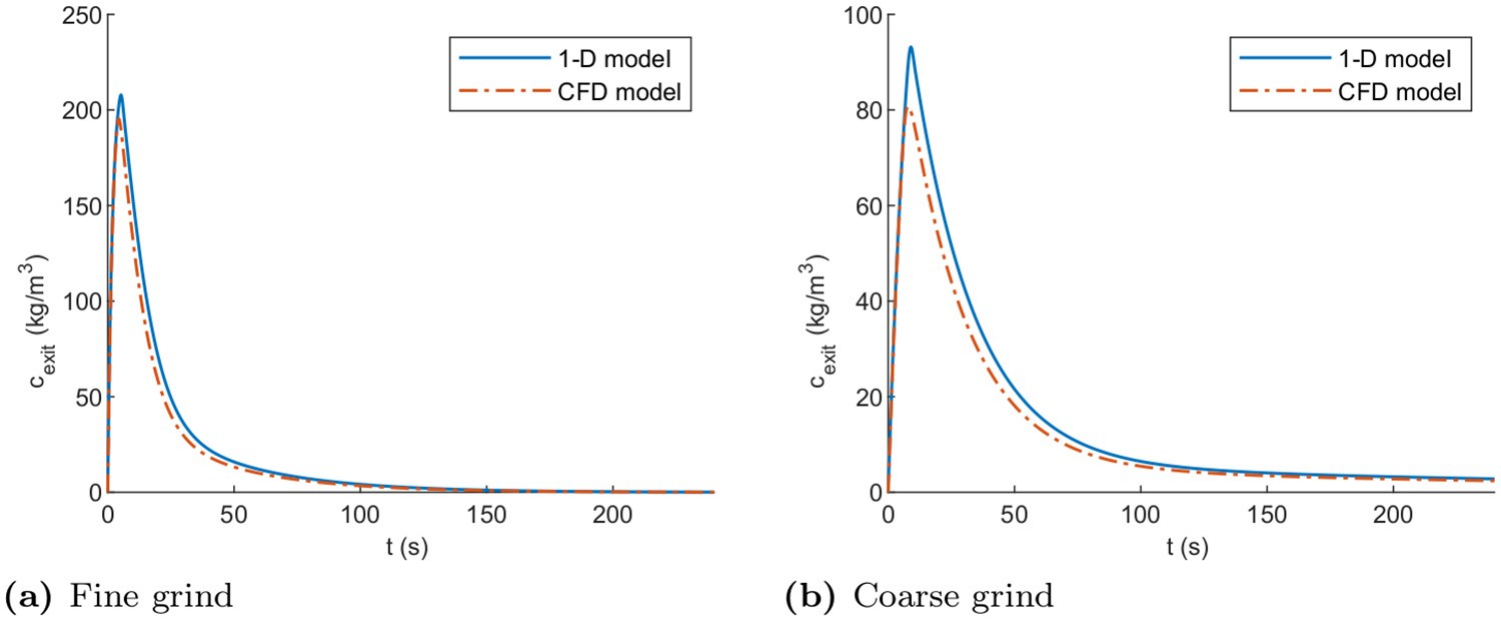
Numerical simulations for conical case. Comparison of numerical models of coffee extraction in the conical geometry using the two grain model for the fine and coarse grinds. Parameters listed in Table 2.

**Fig 9:**
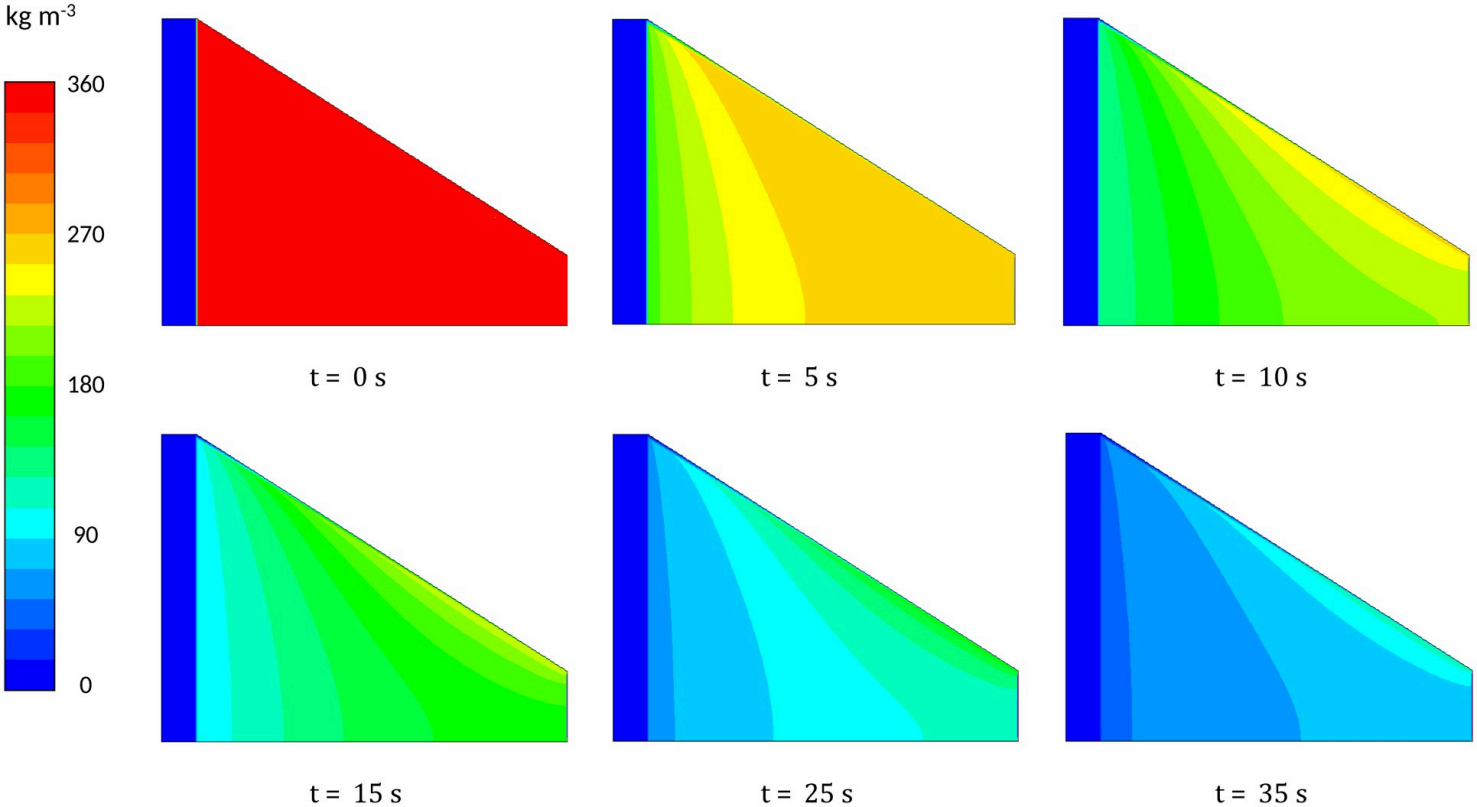
Time evolution of soluble coffee. Time sequence of the remaining soluble coffee concentrations in the coffee bed using the CFD model for the fine coffee grind.

The primary motivation behind modelling the flow pattern in coffee beds is to quantify the impact this has on the extraction uniformity of the bed. Fig 8(a) and 8(b) show the outlet concentration from the conical simulations is actually quite similar to the cylindrical case over the course of extraction. This is the most accessible data from experiment and usually used alone to evaluate extraction (apart from tasting). The spatially resolved CFD model allows us to consider the local extraction yield within the bed as influenced by the non-uniform flow in the bed. Fig 9 shows a time sequence of the remaining soluble content in the coffee grains as extraction progresses. This is a weighted sum of the remaining amounts of coffee in the small and large grains. Depending on preference this can be represented as the local extraction yield % more commonly used by baristas. It is clear here that, while the outlet concentration levels may be similar for both cylindrical and conical geometries, the extraction pattern in the bed may be very different. More detail on these calculations is included in appendices and. Once a particular grind and bed is characterised, the models developed here provide a useful tool to assess the extraction uniformity of the system under different operating and design configurations.

### Extraction uniformity and the coffee brewing control chart

One important question that simulations can address which is hard to measure experimentally is the extent to which grind size distribution and geometry promotes non-uniform extraction. In experimental measurements, the total dissolved solids in the final brew are measured by refractometry and the averaged extraction is calculated from the initial mass of coffee grains and final mass of the brewed coffee. However, it is reasonable to expect that two coffees with the same total dissolved solids and overall extraction yield but with different degrees of extraction uniformity will taste very different. Thus, the simulations reported here allow the investigation of a new property affecting coffee quality: the distribution of extraction yield.

The typical bimodal grind size distribution will be responsible for non-uniform extraction. The difference in size between coarse and fine grains will result in different extraction kinetics. Fines have a larger surface to volume ratio than coarse grains. This will result in faster extraction kinetics. Thus, extraction will be non-uniform in the sense that the extraction yield for fine grains will be different to the extraction yield for coarse grains.

The geometry of the bed is also a factor in determining the inhomogeneity of extraction in flow-through brewing. Fresh water will encounter the uppermost part of the coffee bed first which will promote a vertical extraction gradient. The taper of the bed will also be important. In coffee beds which are cone shaped or conical sections, volume conservation will ensure flow increases as cross section decreases. This will result in the fluid spending less time in contact with the coffee grains where the cross section is smaller. This will also promote a vertical gradient in extraction in the same direction as described previously: with the strongest extraction at the top of the bed and weakest extraction at the bottom. Finally geometry could also, potentially, cause complex flow patterns such as recirculation or dead zones. In these regions fluid would have a very long residence time and extraction would become weak as the fluid would saturate with coffee and remain in residence. (However, these type of complex flow patterns are not observed in the geometries simulated here).

A number of additional factors could also cause flow inhomogeneity. However, these cannot be simulated using the methodology described here, but are potentially accessible to CFD simulation. The microstructure and thus the permeability of the coffee bed is assumed to be uniform in the simulations. If the microstructure was inhomogeneous, e.g. due to fines segregating or poor tamping, then the flow rate and thus extraction rate would also be non-uniform. Incomplete wetting of the coffee bed and non-uniform delivery of water could also promote an uneven extraction.

**Fig 10:**
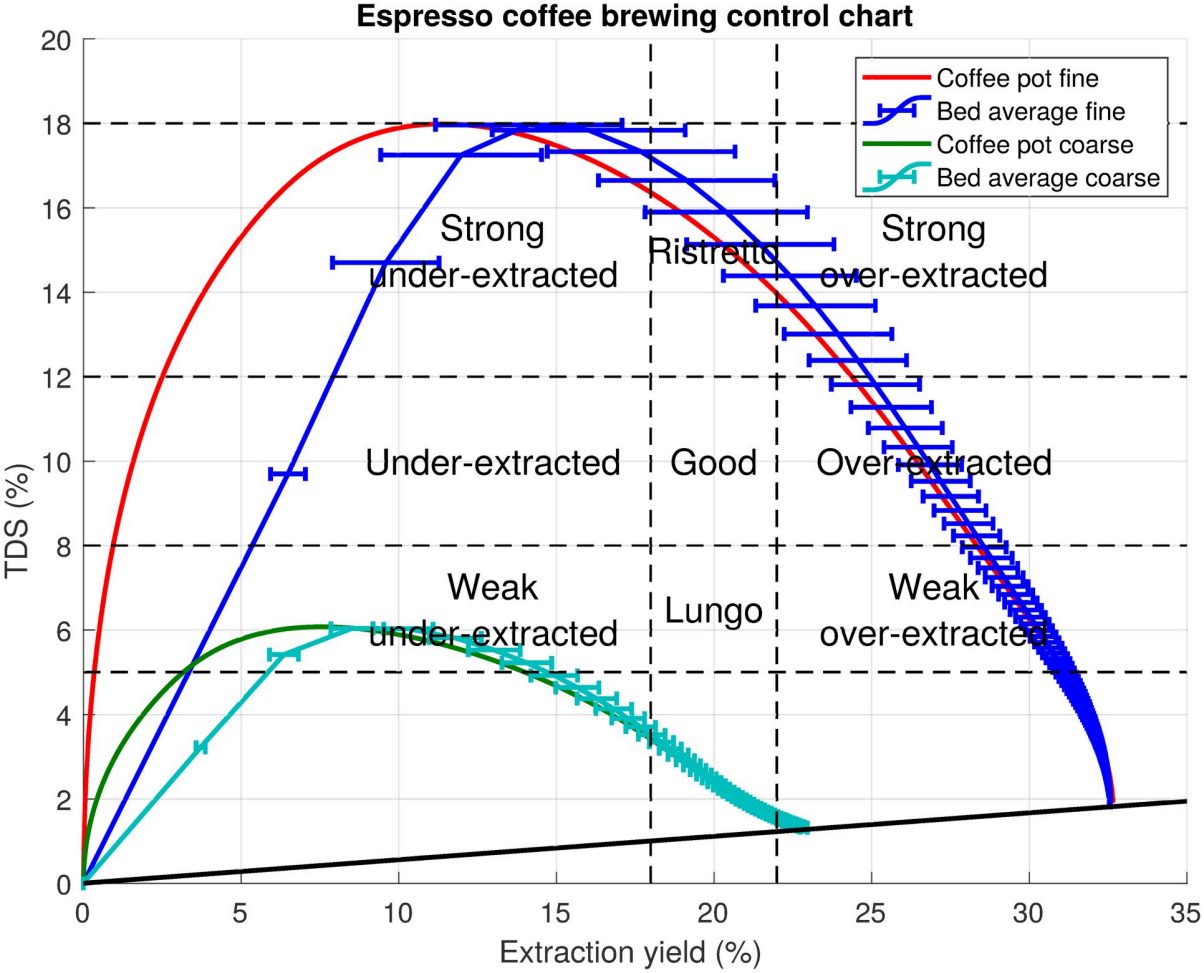
Espresso control chart with extraction uniformity. Coffee brewing control chart for espresso strength preferences. The espresso strength categories are adopted from ref. [10].

To illustrate the point, results for both fine and coarse grind distributions are plotted on the standard coffee brewing control chart in Fig 10. The trajectories are based on the results from the cylindrical brewing chamber for which there is experimental data. The reference concentration at a given time is that in the coffee pot (that is the coffee solution which has left the bed). Usually, just the final point of bed averaged extraction yield and coffee strength are plotted on the chart to evaluate coffee quality. The chart presented here, shows the brew strength vs. extraction yield, as calculated from the mass concentration in the coffee pot at a given time. The trajectories for both the fine and the coarse grinds are plotted. This corresponds to the time sequence of extraction yield and brew strength values during extraction. The new addition is a second curve of the concentration in the coffee pot vs. the average extraction yield of the grains in the bed. The horizontal error bars representing the standard deviation of the extraction yield within the bed are included to quantify the variability of extraction yield. The wider the error bars the more variation there is in extraction level within the bed. Animations showing the time evolution of brew strength and extraction yield on the chart are included as supplementary material.

At the beginning of extraction the extraction yield is zero. As we assume an initial concentration of zero in the water between the grains, the concentration value exiting the bed also starts at zero. In reality (and if extraction during water infiltration was modelled) the initial concentration would be very high and would drop over time as extraction proceeds. In the cylindrical case, the extraction variability arises between the coffee near the bed inlet (highest extraction yield) and the coffee near the bed outlet (lowest extraction yield). As extraction continues, this gap grows to a maximum, before decreasing again as the point where coffee solubles are exhausted is approached. The time taken to reach the maximum concentration level is very short, with a long tail of dilution as the extraction rate slows. This can be observed in Fig 10 as the error bars are evenly spaced in time. The experimental conditions in this paper are most similar to espresso brewing, though the brew ratio used in both experiments is more suitable for drip filter brewing. Thus, as expected, the final brew is highly overextracted (at least for the fine grind) but at a reasonable concentration for drip filter coffee. Based on the extraction curves we note that, for the strength classifications considered here, neither coarse nor fine grind would attain the ideal brew strength for espresso in the target extraction yield range (18%–22%), although the brewing time to achieve these targets can be identified. The fine grind is too strong, falling into the ristretto range, while the coarse grind is too weak and in fact is close to the desired drip filter brew strength target (1.2%–1.45%). A faster flow rate for the fine grind or a slower flow rate for the coarse grind may give the desired strength. In terms of extraction uniformity, the fine grind shows a large variation of about 5 percentage points in extraction yield level in the target range. In comparison, the coarse grind shows a small variation in extraction yield, consistent with the much smaller concentrations which arise in the water phase near the exit. These lower concentrations have a much smaller influence in limiting the extraction rate, relative to those in the fine grind extraction.

## Conclusions

The work in this paper complements recent studies on the modelling of extraction of coffee from packed beds [7, 11, 30–33, 35]. While recent work has considered a variety of physical models of coffee extraction, physical models of flow have largely been restricted to Darcy’s law in one-dimension, incorporating some porosity dependent permeability term. In this study, the flow behaviour is again modelled using a description similar to Darcy’s law, but the problem is solved using a 2-D axisymmetric model in CFD software. A simple model of extraction incorporating two representative grain sizes and first order extraction kinetics is adopted. The impact of deviations from one-dimensional flow in a cylindrical and conical bed geometry was considered by comparing CFD simulations to one-dimensional flow models. A one-dimensional model for flow in a cone is developed. It is found that the one-dimensional assumption is reasonable in the cylindrical geometry, but significant deviations from one-dimensional flow occur in the conical geometry. This work is the first step in considering more complex two- and three-phase flow models of coffee extraction incorporating water infiltration, bed degassing and grain movement, which may all significantly impact on extraction behaviour.

The impact of flow on extraction is considered using a basic description of extraction which depends on the flow model. The local extraction level within the coffee bed is considered. This is influenced by the local fluid velocity and the local concentration of coffee in the intergranular pores. Extraction maps are presented for the conical geometry as a method to evaluate the extraction uniformity of different designs and brewing conditions. The time evolution of brew strength and extraction yield are plotted on the commonly used coffee brewing control chart. This shows the level of variation of extraction yield within the bed, as well as the brew strength-extraction yield trajectory during brewing.

This work highlights the shortfalls of a naive use of the coffee brewing control chart. Differences in extraction percentages exist in any final cup of coffee due to variations in grind size, as well as differences in extraction throughout the coffee bed as brewing occurs. Other factors such as water composition further complicate the situation. The conclusions herein provide some useful insights into the nature and degree of the local extraction level based on bed geometry and fluid flow. Fig 10 represents a useful step in trying to illustrate these on the original coffee brewing control chart. It identifies that any point on the chart is unlikely to represent a precise extraction percentage but an average of a range of local extraction levels in the bed. The degree of variation of extraction level in the bed will impact the taste of the final beverage.

The models applied here are quite adaptable to new descriptions of the underlying processes. A greater understanding of the chemical dissolution and transport processes taking place in a single coffee grain, right up to the complicated fluid dynamics in the coffee bed, will all further assist the hunt for quality and consistency in speciality coffee brewing. In a wider context, the modelling approaches adopted here are applicable to any operation where the uniformity of extraction or indeed filtration across a porous media domain is of key importance.

## Supporting information

S1 Video Coffee control chart fine grind. (MP4)

S2 Video Coffee control chart coarse grind. (MP4)

S1 Appendix Coffee brewing control chart. (PDF)

S2 Appendix Extraction variation within the bed. (PDF)
