# Supplementary material for: Analysing extraction uniformity from porous coffee beds using mathematical modelling and computational fluid dynamics approaches
Source: PLoS One. 2019 Jul 31;14(7):e0219906. doi: 10.1371/journal.pone.0219906 (PMC6668809; doi:10.1371/journal.pone.0219906)
Supplement: S1 Appendix — (PDF) [file pone.0219906.s003.pdf]

S1 Appendix. Coffee brewing control chart. As outlined in the main text, the coffee brewing control chart is based on two measures, namely the extraction yield and the brew strength. Calculation of the brew strength in the models in this paper requires determination of the mass of brew that has exited the bed at a given time and mass of soluble coffee that has exited the bed at a given time. Assuming the volumetric inflow and outflow are the same, $Q(t)$, the brew mass at time t is given by

$$M_{\text{brew}}(t) = \int_0^t \rho_{\text{brew}}(\tau) Q(\tau) d\tau, \tag{1}$$

where $\rho_{\text{brew}}(\tau)$ is the density of solution exiting the bed at time τ. More explicitly, considering the local Darcy volumetric flux u normal to the surface and the density variation at the exit, this becomes:

$$M_{\text{brew}}(t) = \int_0^t \int_{A_{\text{outlet}}} \rho_{\text{brew}}(\vec{x}, \tau) u(\vec{x}, \tau) dA d\tau, \tag{2}$$

where A_{outlet} is the surface at the outlet and dA is a surface element. In this paper, it is assumed that the brew density is equal to the density of water at that temperature and independent of soluble coffee concentration. Similarly expressions for the mass of soluble coffee extracted into the coffee pot can be derived. The mass of soluble coffee in the coffee pot at time t is given by:

$$M_{\text{coffee}}(t) = \int_0^t c_l(\tau) Q(\tau) d\tau, \tag{3}$$

where $c_l(\tau)$ is the concentration of soluble coffee in solution exiting the bed at time τ. More explicitly, considering the local Darcy volumetric flux u normal to the surface and concentration variation at the exit this becomes:

$$M_{\text{coffee}}(t) = \int_0^t \int_{A_{\text{outlet}}} c_l(\vec{x}, \tau) u(\vec{x}, \tau) dA d\tau. \tag{4}$$

In either case, the coffee concentration on a mass per mass basis can be evaluated using:

$$c_{\text{brew}} = \frac{M_{\text{coffee}}}{M_{\text{brew}}}. \tag{5}$$

The strength in TDS% can be found as:

$$S(t) = c_{\text{brew}} \times 100. \tag{6}$$

Similarly the extraction yield (%) is given by:

$$Y(t) = \frac{100 M_{\text{coffee}}}{M_{\text{bed}}}, \tag{7}$$

where M_{bed} is the mass of the dry coffee bed. It should be clear that the brew strength and the extraction yield are related by a quantity similar to the brew ratio so that:

$$S(t) = \frac{M_{\text{bed}}}{M_{\text{brew}}} Y(t). \tag{8}$$

This is actually slightly different from the brew ratio, since M_{brew} may be different to the mass of water used initially, due to water retained in the grains and the addition of soluble coffee to the solution. Other reasons for small differences in these quantities include initial moisture content in the coffee grains (3–4% [1]) and the existence of small amounts of carbon dioxide in freshly ground coffee grains (1–2% [2]). These effects are neglected in the current work. It is also important to note that the models used here use concentration on a mass/volume basis and so results are converted to mass/mass for display on the coffee brewing control chart, using the appropriate density at the relevant temperature.

References

1. Moroney KM, Lee WT, O'Brien SBG, Suijver F, Marra J. Modelling of coffee extraction during brewing using multiscale methods: An experimentally validated model. Chemical Engineering Science. 2015;137:216–234. doi:10.1016/j.ces.2015.06.003.

2. Smrke S, Wellinger M, Suzuki T, Balsiger F, Opitz SE, Yeretzian C. Time-Resolved Gravimetric Method To Assess Degassing of Roasted Coffee. Journal of agricultural and food chemistry. 2017;66(21):5293–5300.
