# Supplementary material for: Analysing extraction uniformity from porous coffee beds using mathematical modelling and computational fluid dynamics approaches
Source: PLoS One. 2019 Jul 31;14(7):e0219906. doi: 10.1371/journal.pone.0219906 (PMC6668809; doi:10.1371/journal.pone.0219906)
Supplement: S2 Appendix — (PDF) [file pone.0219906.s004.pdf]

S2 Appendix. Extraction variation within the bed The evaluation of extraction yield within the coffee bed requires the calculation of the local extraction yield and the variation of extraction yield across the bed. The local extraction yield (%) for one grain size can be defined by:

$$e_y(\vec{x},t) = 100\frac{\phi_0}{\alpha_s}\frac{c_{s0} - c_s(\vec{x},t)}{c_{s0}}. \tag{1}$$

Similarly for two grain sizes the local yield is:

$$e_y(\vec{x},t) = 100\frac{\phi_0}{1-\phi_v}\left(\frac{\alpha_{s1}}{1-\phi_v}\frac{c_{s01} - c_{s1}(\vec{x},t)}{c_{s01}} + \frac{\alpha_{s2}}{1-\phi_v}\frac{c_{s02} - c_{s2}(\vec{x},t)}{c_{s02}}\right). \tag{2}$$

The generalisation to multiple grain sizes should be clear. The average yield over the bed can be computed using:

$$\frac{1}{V_{\text{bed}}}\int_{V_{\text{bed}}} e_y(\vec{x},t)dV, \tag{3}$$

where V_{bed} is the volume of the bed and dV is a volume element. The standard deviation can be calculated using a similar volume weighted standard deviation.
